# Ultrasound-Guided Two-Point Transversus Abdominis Plane in Adult Horses: An Experimental Study Assessing Wall Sensitivity

**DOI:** 10.3390/ani16213401

**Published:** 2026-10-28

**Authors:** Francisco Medina-Bautista, Chiara Di Franco, Irene Nocera, María del Mar Granados, Juan Morgaz, Angela Briganti

**Affiliations:** 1 Department of Veterinary Sciences, University of Pisa, 56124 Pisa, Italy; v52mebaf@uco.es (F.M.-B.); irene.nocera@unipi.it (I.N.); angela.briganti@unipi.it (A.B.); 2 Animal Medicine and Surgery Department, Universidad de Córdoba, 14014 Córdoba, Spain; pv2grmam@uco.es (M.d.M.G.); v92moroj@uco.es (J.M.)

**Keywords:** anesthesia, horse, TAP, lidocaine, ultrasound

## Abstract

Ultrasound-guided transversus abdominis plane blocks may provide somatic analgesia for equine abdominal procedures, but evidence in adult horses remains limited. This prospective, blinded, randomized, paired study evaluated the feasibility, sensory effects, and motor effects of a two-point transversus abdominis plane block in eight healthy, standing, unsedated mares. Each horse received lidocaine 2% (2 mg/kg in 0.1 mL/kg per injection site) on one side and saline on the contralateral side. Responses to standardized mechanical stimulation were assessed at 21 sensory evaluation points per side for 300 min. The block was completed successfully in all horses without visible discomfort or procedural complications. No desensitization occurred on saline-treated sides. Following lidocaine administration, sensory blockade began at a median of 10 min, peaked at 30 min, and lasted a median of 258 (233–260) minutes. Desensitization was significantly greater at the stifle and intermediate horizontal levels than at the coxal tuberosity level. Transient motor impairment occurred in seven of eight horses and was severe in two. The technique produced multi-metameric desensitization, predominantly in the ventrocaudal flank, but limited dorsal coverage and frequent motor impairment may restrict its clinical application. Further dose optimization and clinical safety and efficacy studies are warranted.

## Simple Summary

Pain after abdominal surgery can negatively affect the welfare and recovery of horses. Local anesthetic techniques may reduce pain while limiting the adverse effects associated with pain-relieving drugs administered throughout the body. This study investigated an ultrasound-guided injection technique intended to numb the abdominal wall of standing horses. Eight healthy mares received lidocaine on one side of the abdomen and saline on the opposite side Skin sensitivity was tested for five hours by applying a standardized pinch with a surgical clamp at 21 predefined points on each side of the abdomen and recording whether the horse showed a behavioral or reflex response. Coordination was also assessed. The technique was completed successfully in all horses without visible discomfort or injection-related complications. Loss of skin sensitivity began within 10 min, was greatest after 30 min, and lasted approximately four hours. Desensitization was more extensive in the lower and rear regions of the flank, whereas the dorsal region showed limited coverage. Seven of the eight horses developed temporary coordination problems, including severe impairment in two. This technique can desensitize part of the abdominal wall, but its limited upper coverage and frequent effects on coordination indicate that further studies are needed to refine the dose and injection method and to establish its safety and effectiveness during surgery.

## 1. Introduction

Laparoscopic celiotomy offers several advantages in equine surgery [1]. This minimally invasive technique allows clear visualization of abdominal organs while being considered safer than conventional surgery under general anesthesia for both horses and veterinary personnel [2].

Additionally, laparoscopy is associated with faster recovery, reduced postoperative pain, and lower morbidity and hospitalization rates in veterinary medicine [3,4,5,6]. However, intraoperative and postoperative complications can still occur, with postoperative pain being one of the most common complications, affecting up to 32.7% of cases [7].

Common strategies to alleviate abdominal pain in horses often rely on the administration of systemic analgesics [8]. Although effective, these drugs may produce undesirable side effects such as gastrointestinal hypomotility and postoperative ileus, locomotor excitation or dysphoria, cardiovascular depression prolonged and poor-quality recovery from general anesthesia [9,10,11,12,13,14,15,16]. As an alternative, locoregional techniques using local anesthetics may offer effective analgesia with a reduced risk of systemic adverse effects. Techniques such as paravertebral nerve blocks and local portal infiltration have shown efficacy in controlling intraoperative pain for laparoscopic closure of the nephrosplenic space [17]. Another promising technique is the transversus abdominis plane (TAP) block, an interfascial block that targets the nerves supplying the abdominal wall [18]. The TAP block provides somatic analgesia to the skin, subcutaneous tissue, muscle layers, parietal peritoneum, prepuce, and mammary gland, as demonstrated in humans, cats, and dogs [19,20,21,22]. In adult horses, data remain limited to two cadaveric studies [23,24] and one experimental study [25]. Baldo et al. [23] demonstrated extensive spread following a high-volume (0.5 mL/kg) TAP injection but highlighted the potential risk of femoral nerve involvement which could adversely affect the motor function. In contrast, Freitag et al. [24] described a more ventral approach targeting the lower abdominal wall, which may provide limited coverage of the more dorsally located surgical portals commonly used during equine abdominal procedures [26]. Similarly, Küls et al. [25] evaluated a lower abdominal TAP approach and reported reduced responses to pinprick stimulation; however, the block failed in two out of six animals, suggesting variability in cutaneous desensitization. Anesthesia denotes loss of sensation, whereas analgesia specifically denotes absence of pain in response to normally painful stimulation [27]. In the present study, cutaneous desensitization describes a reduced or absent response to cutaneous stimulation. The objective of this study was to assess the feasibility of an ultrasound-guided two-point TAP block in horses and to characterize the extent and duration of abdominal cutaneous desensitization and any associated and motor effects.

## 2. Materials and Methods

### 2.1. Study Design

A prospective, blinded, randomized, controlled experimental trial with a paired design was conducted in accordance with the ARRIVE Essential 10 reporting guidelines [28]. Two experienced operators (I.N. and C.D.F.), both trained in the TAP block technique, performed all injections. Each operator performed the two TAP injections on a fixed abdominal side in all eight horses, while the other operator performed the injections on the contralateral side. Each horse received lidocaine (LID) on one side and saline (SAL) on the opposite side. Treatment allocation was randomized using paper slips placed in a box (eight labelled “LID” and eight labelled “SAL”), drawn by an independent individual not involved in the study. Once treatment was assigned to one side, the contralateral side received the alternate treatment. Treatment allocation was balanced between operators: each administered lidocaine in four horses and saline in four horses, resulting in 16 injections per operator (eight with lidocaine and eight with saline). Both operators were blinded to treatment allocation before preparation of the injectate and remained blinded throughout the injection procedures.

### 2.2. Animals

Eight Italian Trotter mares were included in the study. Mean body weight was 513.4 ± 45.2 kg and mean age was 18.1 ± 5.5 years, while median body condition score was 6/9 (4–6). A cadaver from an 18-year-old Italian Trotter gelding weighing 450 kg had previously been used for the preliminary anatomical dissection. Mares were considered eligible if they were adults, had a body condition score between 4 and 6 on a nine-point scale, and had no reported history of abdominal or musculoskeletal pain or trauma involving the abdominal wall or hindlimbs. All horses were considered clinically healthy based on physical examination performed prior to the study. Temperament was assessed before the beginning of the study according to the criteria described by [29]. Five horses (62.5%) were classified as calm and three (37.5%) as nervous. All horses were owned by the University of Pisa and housed at its Veterinary Teaching Hospital, where they were maintained under standard husbandry conditions, receiving hay and pellets with ad libitum access to water. Ethical approval for all procedures was granted by the Italian Ministry of Health (authorization no. 211/2025-PR). Private-owner informed consent was not applicable because all horses were owned by the University of Pisa. The predefined exclusion criteria were failure to clearly identify the anatomical landmarks during ultrasound scanning or failure to visualize separation of the fascial planes during injection (hydrodissection). None of the eight horses met these exclusion criteria, and all were retained in the analysis. No deviations from the planned experimental protocol occurred. All scheduled observations were collected, and there were no missing data.

The number of horses enrolled was based on a priori sample size calculation using a single-proportion precision-based approach, aiming to estimate an expected response rate of 90% for successful desensitization at the imaginary equidistant between the coxal tuberosity and the stifle in the lidocaine (LID) group, with a 95% confidence level and a precision of ±15% [30]. The sample size was calculated using the formula for estimation of a proportion (***n*** = ***Z*^2^** × ***p*** × [**1** − ***p***]/***d*^2^**), where *Z* corresponds to the standard normal deviate for the desired confidence level, *p* is the expected proportion, and *d* is the desired precision. Each horse received four injections: two right caudal to the last rib and right cranial to the coxal tuberosity on one randomly assigned side with lidocaine (LID group), and two at the same levels on the contralateral side with saline (SAL group).

### 2.3. TAP Injections

**Figure 1:**
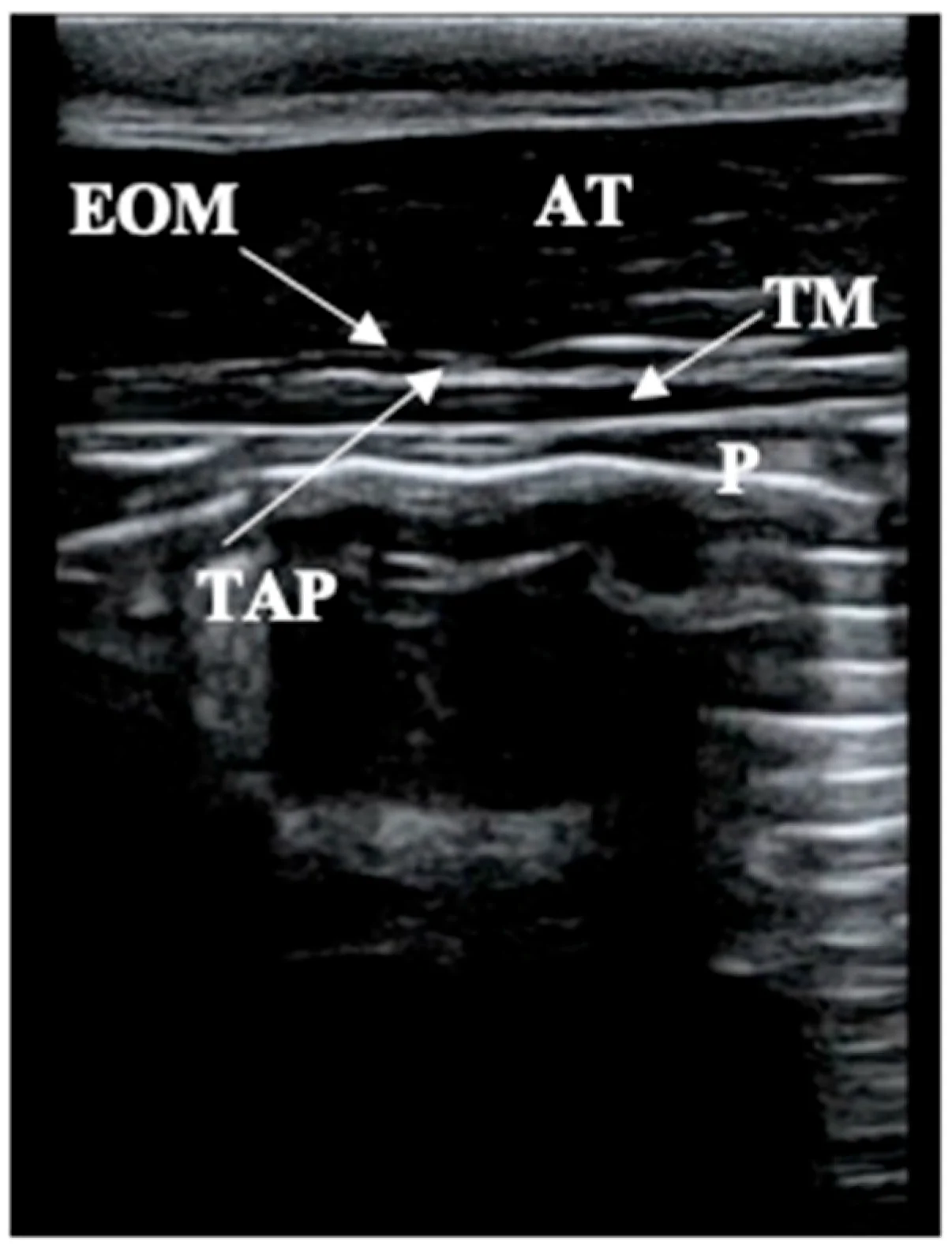
Ultrasound image of the caudal transversus abdominis plane (TAP) injection site. The ultrasound probe was positioned immediately cranial to the coxal tuberosity, transverse to the spine, with the probe marker directed dorsally. At this level, only the external abdominal oblique muscle (EOM) and the transversus abdominis muscle (TM) are visible. The target interfascial plane for local anesthetic injection is located between both muscles. P: Peritoneum (hyperechoic line under TM). AT: Adipose tissue.

**Figure 2:**
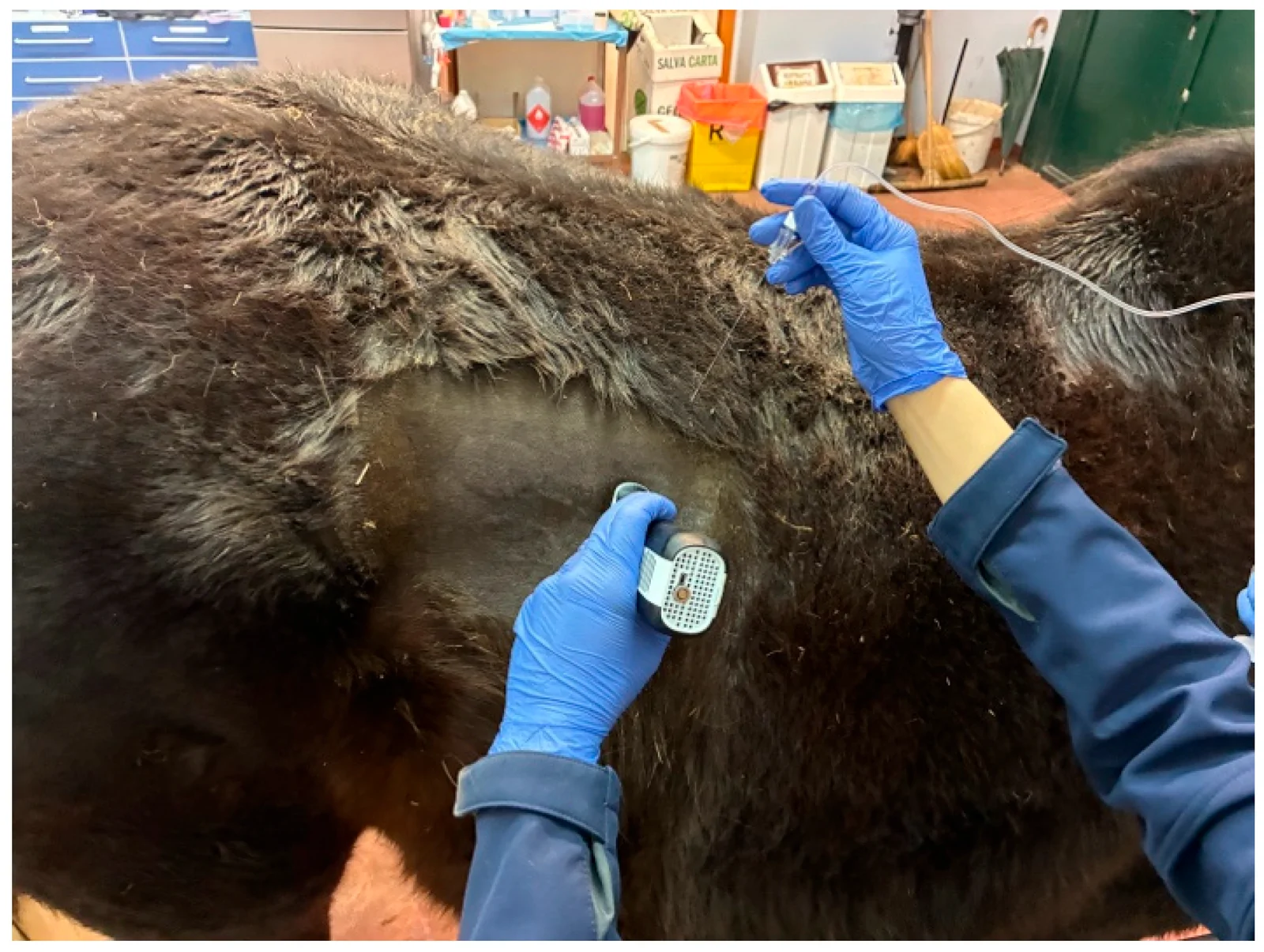
Positioning of the ultrasound probe for the cranial transversus abdominis plane (TAP) injection site. The probe was positioned immediately caudal to the last rib, transverse to the spine, at the level of the coxal tuberosity, with the probe marker directed dorsally.

**Figure 3:**
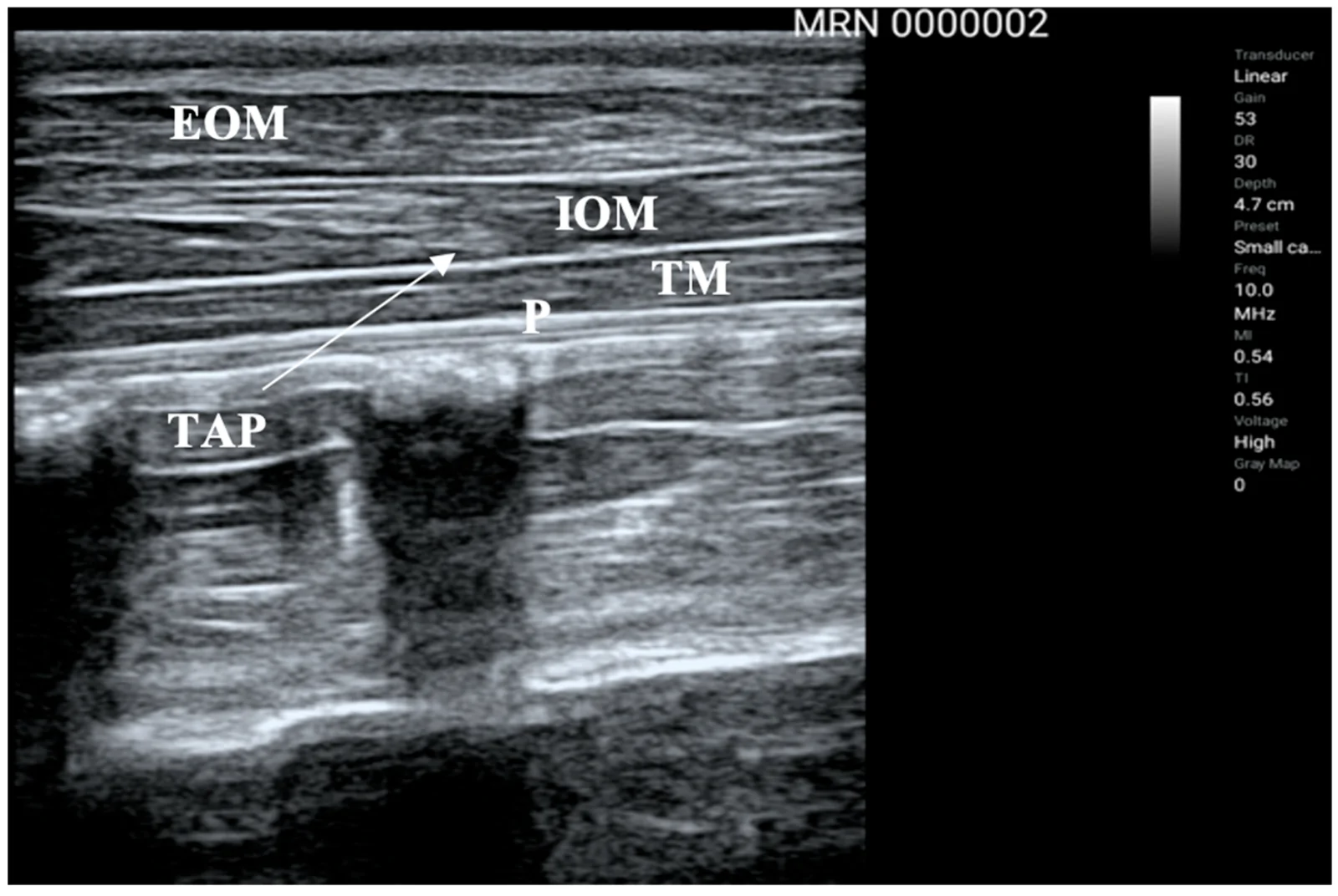
Ultrasound image of the cranial transversus abdominis plane (TAP) injection site (arrow) obtained with the ultrasound probe positioned immediately caudal to the last rib, transverse to the spine, at the level of the coxal tuberosity, with the probe marker directed dorsally. At this level, the external abdominal oblique muscle (EOM), internal abdominal oblique muscle (IOM), and transversus abdominis muscle (TM) are identified. The target transversus abdominis plane (TAP) is located between the IOM and TM. P: Peritoneum (hyperechoic line under TM).

The area between the last intercostal space and the coxal tuberosity was clipped and aseptically prepared. For the first point (caudal injection site), the probe (ASUS Handheld Ultrasound Solution LU700L, Vet 5–10 MHz; Dramiński S.A., Sząbruk, Poland) was placed just cranial to the iliac crest transversally to the spine with the marker dorsally. At this level, only the abdominal external oblique and transversus abdominal muscle could be visualized (Figure 1). For the second point (cranial injection site), the probe was placed just caudal to the last rib transversally to the spine with the marker dorsally and at the height of the coxal tuberosity (Figure 2). In this point both abdominal external and internal abdominal oblique muscles and the abdominal transversus muscle could be visualized (Figure 3).

**Figure 4:**
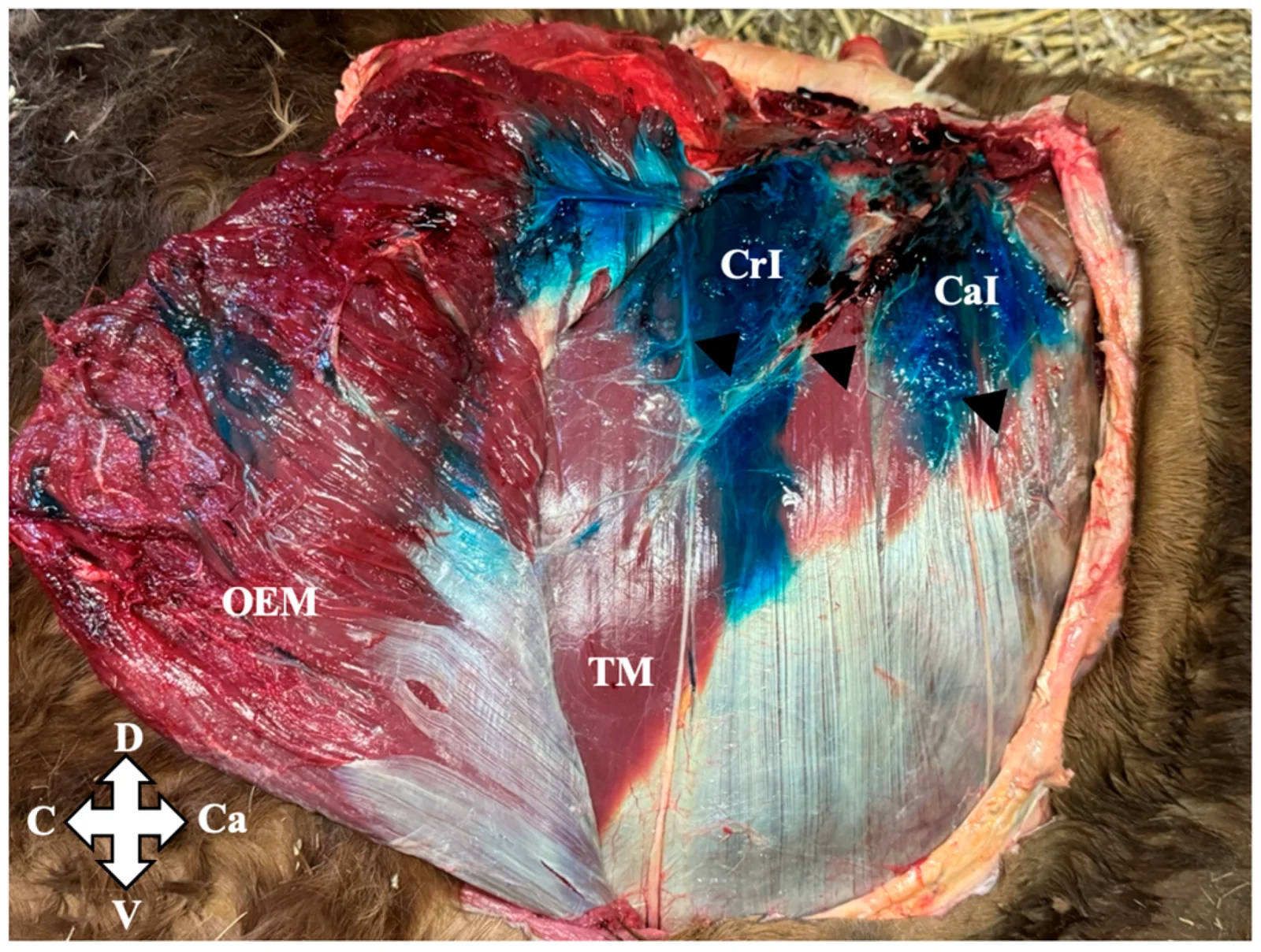
Cadaveric dissection in a horse of the injectate volume used for the ultrasound-guided transversus abdominis plane (TAP) block. Two TAP injections [cranial (CrI) and caudal (CaI)] were performed on each hemilateral abdomen (four injections per cadaver), using 0.1 mL/kg per injection site. Following reflection of the external abdominal oblique muscle (EOM), methylene blue was consistently identified within the transversus abdominis plane overlying the transversus abdominis muscle (TM), staining the ventral rami (arrowhead) confirming appropriate interfascial distribution and supporting the injectate volume selected for the in vivo study. D: dorsal; V: ventral; Cr: cranial; Ca: caudal.

Once both injection sites were identified, the skin and subcutaneous tissues were desensitized using 50 mg of lidocaine (Lidocaina 2%, Ecuphar, Milan, Italy) per site. After 5 min, a 10-cm 20 G echogenic needle (Visioplex, Vygon, Padua, Italy), primed and connected to a 60 mL syringe (Monoject Luer Lock, Cardinal Health, Dublin, OH, USA), was introduced in a dorsolateral-ventromedial direction at approximately 45° to the skin surface, with the bevel facing upwards. In both injections, the needle was addressed to the aponeuroses of insertion of both muscles (Supplementary Material). Throughout the procedure, both the needle shaft and tip were continuously visualized under ultrasound guidance to ensure accurate placement and safe injection. After a negative aspiration test, needle placement was confirmed by the injection of 2 mL of local anesthetic to visualize hydrodissection of the TAP between the abdominal internal oblique and transversus muscles at the cranial injection site, and between the abdominal external oblique and transversus muscles at the caudal injection site. A volume of 0.1 mL/kg per site containing 2 mg/kg of lidocaine (LID group) or 0.9% saline solution (Sodium Chloride 0.9%, B.Braun, Melsungen, Germany) (SAL group) was then injected slowly over 2 min. The cumulative lidocaine dose per horse was 4 mg/kg from the two TAP injections plus 200 mg for skin and subcutaneous infiltration at the four injection sites, equivalent to approximately 4.39 mg/kg at the mean study body weight (513.4 kg). A volume of 0.1 mL/kg per injection site was selected based on cadaveric dissections (unpublished data), which demonstrated consistent spread within the target interfascial plane while minimizing dorsal extension toward the lumbar plexus (Figure 4).

Injection times, defined as the duration from initial contact of the ultrasound probe with the patient to completion of the injection, were recorded. Each horse’s response during administration of the TAP injectate was assessed by the operator performing the injection. Responses were recorded as “Yes” (signs of discomfort) or “No” (no reaction) based on observable behaviors, including panniculus reflex, skin twitching, visible increase in muscle tone, tail movement, leg withdrawal, or stepping away. All procedures were performed in standing, unsedated horses.

### 2.4. Noxious Mechanical Stimulation and Sensory and Motor Assessment

Metamers from Th17 to L5 were identified by palpation and marked for efficacy assessment based on anatomical landmarks. Block efficacy in the flank region was evaluated using a standardized noxious mechanical stimulation test to assess both craniocaudal (CC) and dorsoventral (DV) spread [30].

Craniocaudal spread was assessed across seven consecutive metamers (Th17, Th18, and L1–L5), while DV spread was assessed using three horizontal assessment lines located at the level of the coxal tuberosity, equidistant between the coxal tuberosity and the stifle, and at the level of the stifle. This resulted in 21 sensory evaluation points (SEPs) per side (7 metamers × 3 horizontal assessment lines), and 42 SEPs per horse. Noxious mechanical stimulation was performed using a Kelly clamp (Straight, 14 cm; GIMA S.p.A., Gessate, Italy). A single blinded trained investigator (F.M.-B.) carried out all assessments. For each test, the clamp was applied perpendicularly to the skin and closed to the first locking position. A response was considered positive if any of the following signs were elicited: panniculus reflex, skin twitching, visible increase in muscle tone, tail movement, leg withdrawal, or stepping away. In the absence of a response, the point was classified as negative. Only one stimulation was performed per point at each time. Assessments were performed in random order across metamers, except at baseline (TB), which followed a fixed sequence. Baseline testing occurred 15–30 min before the first TAP injection. During baseline assessment (TB), the left thoracolumbar region was tested first, starting at L5 and proceeding cranially to Th17. The right side was tested in the same manner. Noxious mechanical stimulation responses were then recorded at predefined time points selected to capture the onset and duration of lidocaine-induced cutaneous desensitization: 5 (T5), 10 (T10), 15 (T15), 30 (T30), 60 (T60), 90 (T90), 120 (T120), 150 (T150), 180 (180), 210 (T210), 240 (T240), 270 (T270) and 300 (T300) minutes after each injection. When a specific assessment point tested positive in two consecutive evaluations, it was not reassessed in subsequent time points and was considered positive for the remainder of the study. The second TAP injection was administered immediately after completing the first. Onset of sensory blockade was defined as the time from completion of the TAP injection to the first negative response to noxious mechanical stimulation at any sensory evaluation point (SEP). Duration of sensory blockade (minutes) was defined as the time interval from the first observed negative response at any SEP until no desensitized SEPs remained. Motor impairment was evaluated subjectively by the same investigator (F.M-B.) at each time point using a 4-point ordinal scale (4 = severe; 3 = moderate; 2 = mild; 1 = none) [31].

### 2.5. Statistical Analysis

Statistical analyses were performed using IBM SPSS Statistics Version 29.0.2.0 (IBM Corp., Armonk, NY, USA). Continuous variables recorded once per horse, including age, body weight, body condition score, injection times, onset and duration of sensory blockade, and onset and duration of motor impairment, were assessed for normality using the Shapiro–Wilk test and visual inspection of histograms and Q–Q plots. Normally distributed variables were expressed as mean ± SD, whereas non-normally distributed variables were reported as median (25th–75th percentiles). Categorical and ordinal variables, including temperament, response to injection, and maximum motor impairment grade, were summarized as absolute frequencies and percentages. No inferential statistical comparisons were performed for these descriptive variables because these outcomes were considered descriptive and exploratory in nature. Responses to noxious mechanical stimulation at each SEP were recorded as binary outcomes (positive or negative). For each horse, time point, and horizontal assessment line, the number of desensitized sensory evaluation points (SEPs) along the craniocaudal axis was calculated by summing the negative responses to noxious mechanical stimulation across the seven SEPs corresponding to metamers Th17, Th18, and L1–L5. This generated one desensitization score per horizontal assessment line at each time point, ranging from 0 to 7, where 0 indicated no desensitized SEPs and 7 indicated absence of response at all evaluated SEPs. Only data from the LID group were included in the inferential analysis, as no desensitization was observed in saline-treated sides throughout the study period. Data were analyzed using a linear mixed model to account for repeated measurements obtained from the same horse over time. Dorsoventral spread, time, and their interaction were included as fixed effects, while horse was included as a random intercept. Fixed effects were evaluated using F-tests and are reported as F statistics with corresponding degrees of freedom and associated *p* values. When significant main effects or interactions were identified, estimated marginal means were compared using Bonferroni-adjusted pairwise comparisons. Statistical significance was set at *p* < 0.05.

## 3. Results

The TAP block was successfully performed in all horses, and no complications associated with block placement were observed. Median injection times are shown at Table 1. No horse showed signs of discomfort during injection.

**Figure 5:**
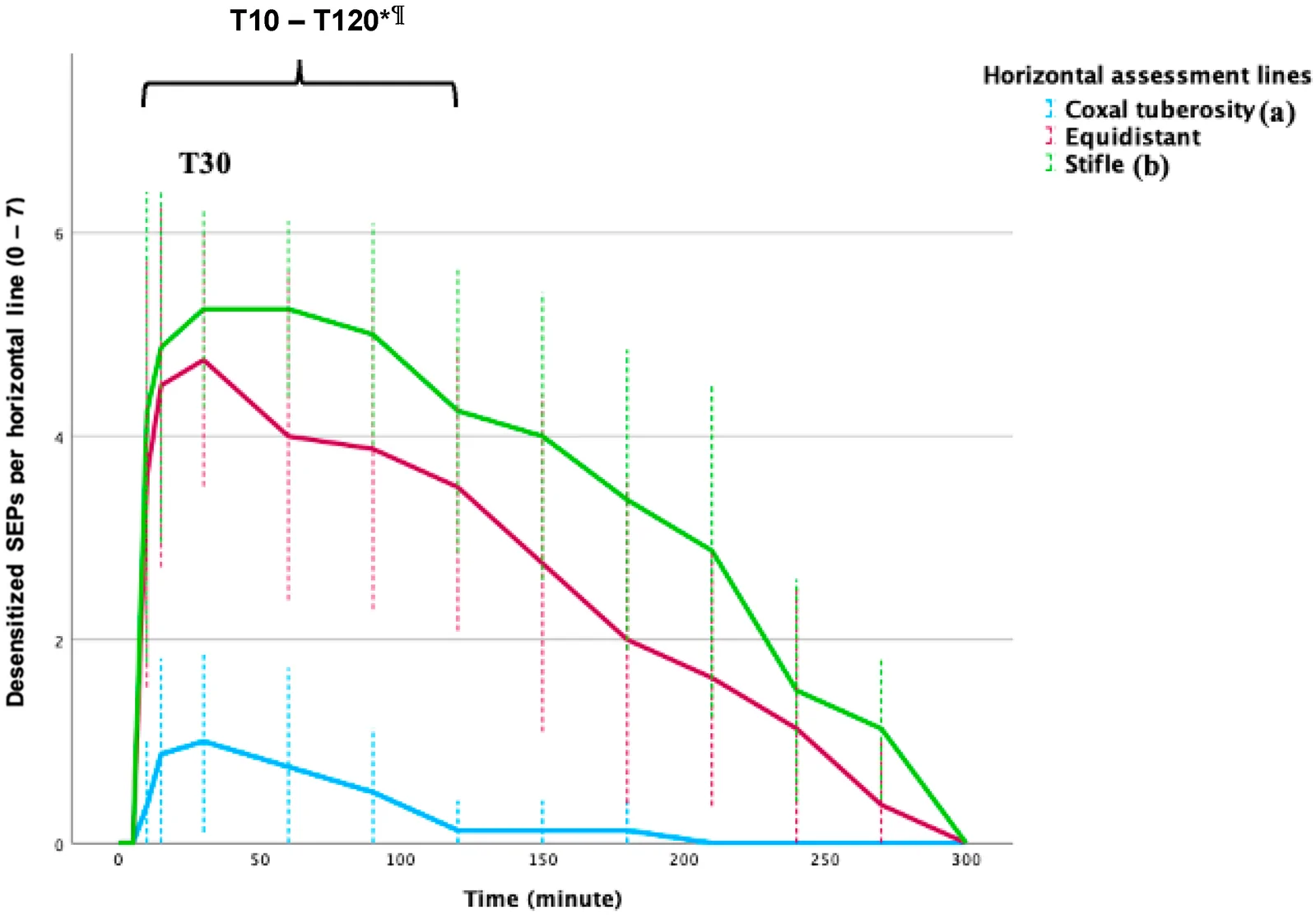
Graph line showing the temporal evolution of the mean number of desensitized sensory evaluation points (SEPs) along the craniocaudal axis (Th17, Th18, and L1–L5) at the three horizontal assessment lines following transversus abdominis plane (TAP) block in the lidocaine-treated side (LID group). Horizontal assessment lines corresponded to the level of the coxal tuberosity, equidistant line between the coxal tuberosity and the stifle, and the level of the stifle. Values are presented as mean ± 95% confidence intervals. Horizontal assessment lines not sharing a letter (a,b) differ significantly (Bonferroni-adjusted pairwise comparisons, *p* < 0.05). At baseline (T0), the mean number of desensitized SEPs was zero for all three horizontal assessment lines; therefore, the corresponding values overlap with the *x*-axis. * Significant main effect of time compared with baseline (TB) from 10 (T10) to 120 (T20) minutes (*p* < 0.05). **^¶^** Maximum overall desensitization.

Sensory blockade was first detected after a median of 10 min (10–10) following block placement. The duration of sensory blockade was 258 min (233–260). The number of desensitized SEPs increased significantly from baseline at T10 and remained significantly greater than baseline through T120. The highest estimated marginal means for the effect of time were observed at T30 after injection, with 1.83 desensitized SEPs per horizontal assessment line. Thereafter, the extent of desensitization progressively decreased, and no significant differences from baseline were detected from T150 onwards (Figure 5). The number of desensitized SEPs was significantly influenced by horizontal assessment line (F_2_,_623_ = 42.003, *p* < 0.001) and time (F_13_,_623_ = 8.527, *p* < 0.001). However, the interaction between horizontal assessment line and time was not statistically significant (F_26_,_623_ = 1.426, *p* = 0.079).

**Figure 6:**
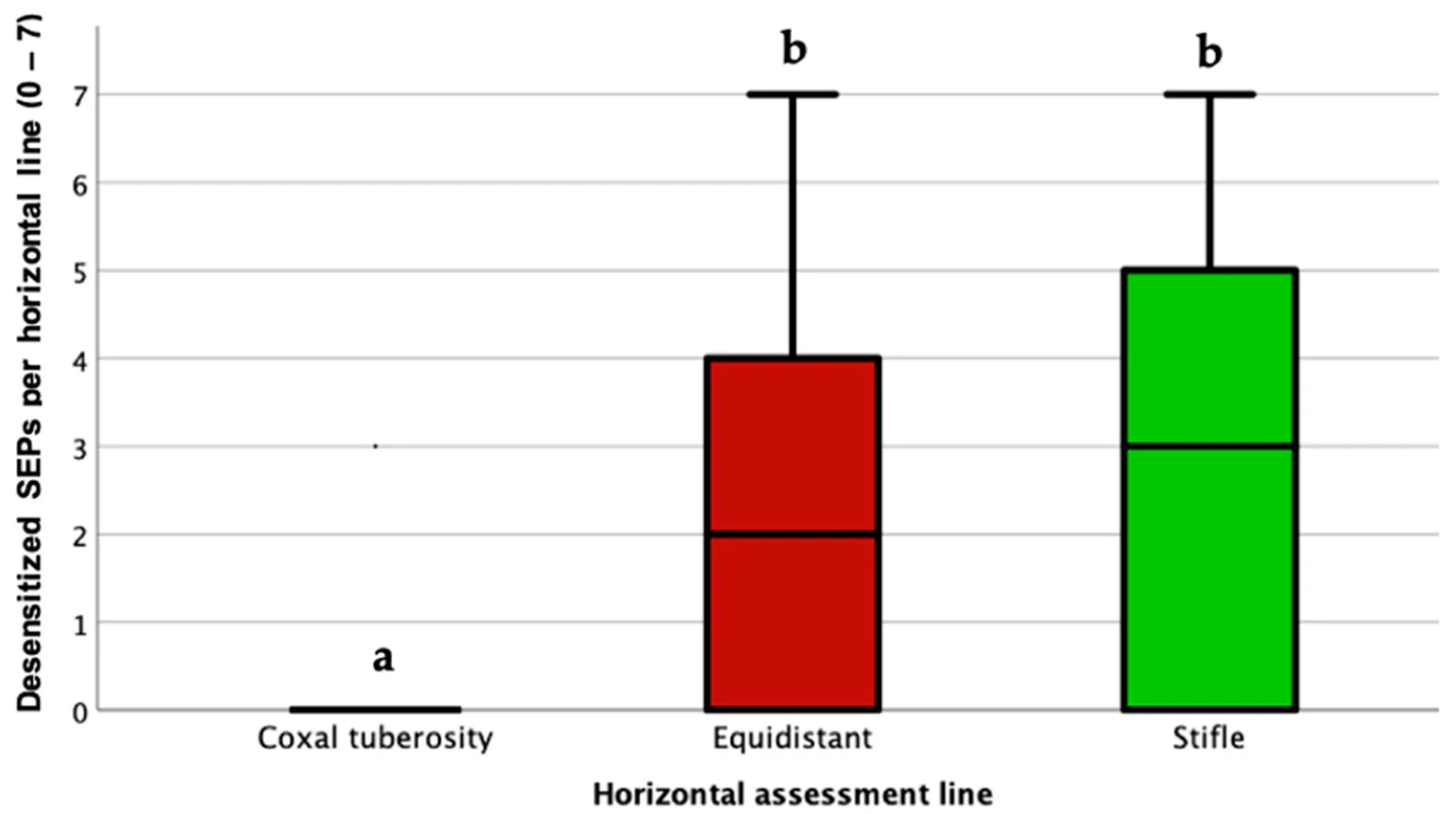
Boxplot showing the distribution of the number of desensitized sensory evaluation points (SEPs) along the craniocaudal axis (Th17, Th18, and L1–L5) at each horizontal assessment line (coxal tuberosity, equidistant, and stifle) in lidocaine-treated sides (LID). Data from all horses and time points [baseline (TB) to 300 (T300) minutes after block] were pooled. Each horizontal assessment line included seven SEPs, yielding a possible score ranging from 0 to 7. Boxplots represent the median (horizontal line), interquartile range (box), and range (whiskers). Horizontal assessment lines not sharing a letter differ significantly (Bonferroni-adjusted pairwise comparisons, *p* < 0.05).

Estimated marginal means showed that the highest number of desensitized SEPs was observed at the stifle level (1.49; 95% CI: 1.17–1.81), followed by the equidistant horizontal line (1.15; 95% CI: 0.83–1.46), whereas the lowest values were recorded at the coxal tuberosity level (0.14; 95% CI: −0.18 to 0.46). Pairwise comparisons revealed significantly fewer desensitized SEPs at the coxal tuberosity level compared with both the equidistant horizontal line (mean difference = −1.01, *p* < 0.001) and the stifle level (mean difference = −1.35, *p* < 0.001). No significant difference was detected between the equidistant horizontal assessment line and the stifle level (*p* = 0.076) (Figure 6).

**Figure 7:**
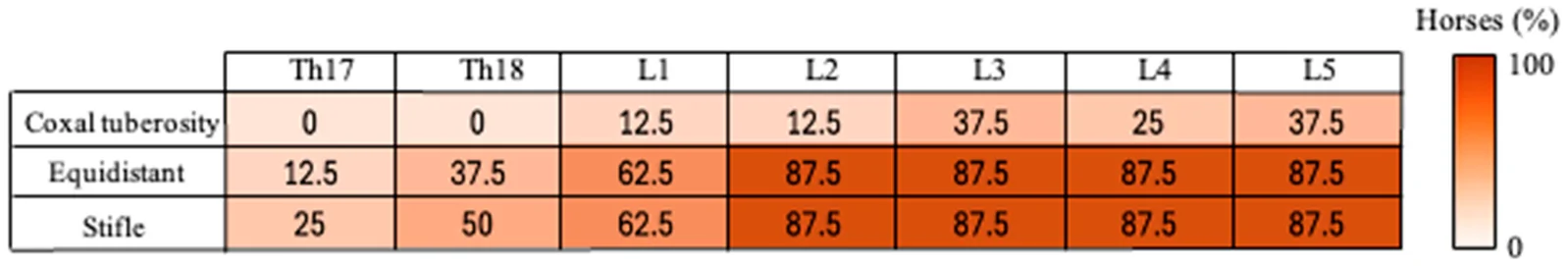
Heat map showing the proportion of horses with desensitization at each sensory evaluation point (SEP) 30 min (T30) after lidocaine injection (LID group), corresponding to the peak effect of the block. Columns represent metamers (Th17, Th18, and L1–L5), whereas rows correspond to the three horizontal assessment lines (coxal tuberosity, equidistant line, and stifle level). Values within each cell represent the percentage of desensitized horses out of 8 total horses. Darker orange shading indicates greater desensitization.

The spatial and temporal distribution of desensitization across the flank region is summarized in Figure 7.

Post-block motor impairment developed after a median of 10 min (10–10) and lasted for 260 min (163–260). The highest motor impairment grade observed was moderate in three horses (37.5%), mild in two horses (25%), severe in two horses (25%), and absent in one horse (12.5%). Motor impairment was most pronounced between 10 and 60 min after block placement and progressively improved thereafter, with complete resolution in all horses by the end of the study period.

## 4. Discussion

The present study demonstrates that an ultrasound-guided two-point transversus abdominis plane (TAP) block tailored for flank celiotomy is feasible in standing, unsedated horses and provides consistent multi-metameric desensitization of the lateral abdominal wall following lidocaine 2% administration. The technique was successfully performed in all horses, with short and consistent injection times, no procedural complications, and no signs of discomfort during block placement. Sensory blockade developed rapidly, with onset occurring within 10 min, reaching maximal effect at 30 min, and persisting for approximately 4 h. However, desensitization was not uniformly distributed, being significantly greater at the equidistant and stifle levels compared with the level at the coxal tuberosity. In addition, transient motor impairment was observed in most horses, highlighting potential safety concerns that warrant further investigation.

The short injection times observed in this study suggest that this TAP approach could be practical, feasible and reproducible in clinical settings. Median injection times ranged between 2 and 3 min per injection site, indicating that bilateral four-point TAP block placement can be completed rapidly without substantially prolonging preparation time. These findings are consistent with previous equine ultrasound-guided interfascial plane block studies, including TAP and erector spinae block (ESP) investigations, in which injection times of approximately 2–3 min per site have also been reported [24,30]. The fact that all procedures were performed in standing, unsedated horses further support the practicality of the technique and enhances its potential applicability for standing procedures [2]. Moreover, the absence of visible discomfort during injection suggests that the technique was well tolerated.

The sensory findings support the ability of this TAP approach to provide clinically relevant somatic desensitization of the flank region. Block onset occurred consistently at 10 min, and although significant desensitization persisted for 120 min, residual sensory blockade remained evident for approximately 258 min. Maximum desensitization occurred at 30 min, after which sensory blockade progressively declined. This likely reflects a gradual reduction in blockade intensity rather than abrupt loss of effect. Both the onset and duration observed in the present study are comparable to those previously reported in horses following thoracolumbar ESP block with lidocaine 2% [30] and TAP with low concentrated bupivacaine (0.125%) [25]. Lidocaine was selected to characterize the feasibility, sensory distribution, duration, and motor effects of this TAP approach, rather than to reproduce a postoperative analgesic protocol using a longer-acting agent. Nevertheless, residual cutaneous desensitization remained detectable for approximately 258 min despite the use of lidocaine. The duration of sensory blockade following interfascial plane blocks is thought to depend not only on the physicochemical properties of the local anesthetic but also on the anatomical characteristics of the fascial compartment. Factors such as tissue compliance, elasticity, needle design, fascial sliding properties, vascularity, and the resulting rate of systemic absorption may influence local anesthetic spread and clearance, thereby affecting block coverage and duration [32,33,34,35]. Evidence from equine rectus sheath blocks is consistent with a potential depot effect of the interfascial compartment. Ishikawa et al. [36] reported abdominal midline antinociception lasting at least 8 h following bupivacaine administration. Subsequently, Sakai et al. [37] documented prolonged systemic absorption, with detectable plasma bupivacaine concentrations at 24 h, and concluded that even this sampling period was insufficient to accurately determine the terminal half-life. These findings suggest that the interfascial compartment may act as a drug depot, contributing to sustained local anesthetic exposure. However, this mechanism was not evaluated for lidocaine in the present study.

The spatial distribution of desensitization provides important insight into the spread characteristics of this TAP technique. Desensitization was significantly lower at the coxal tuberosity level than at the equidistant and stifle levels, indicating greater ventrocaudal than dorsocranial spread of local anesthetic. This pattern is consistent with the anatomical course of the ventral branches of the thoracolumbar nerves, which travel between the transversus abdominis and internal abdominal oblique muscles before supplying the abdominal wall [23,24]. Moreover, all blocks in the present study were performed with horses positioned in standing, a factor that may also have influenced injectate distribution. Indeed, Freitag et al. [24] demonstrated that body positioning affects injectate spread, reporting greater cranial distribution when the modified subcostal TAP block was performed in lateral rather than dorsal recumbency, likely reflecting the influence of gravitational forces and fascial plane orientation. The mechanisms of action of interfascial plane blocks and the factors determining their sensory distribution remain incompletely understood [38]. Tissue anisotropy may contribute to the regional differences in cutaneous desensitization observed, with lower desensitization dorsally than in the middle and ventral abdominal regions. Istenič et al. [39] demonstrated preferential distribution of bupivacaine along rather than across muscle fibers in fresh human cadaveric skeletal muscle. The dorsoventral orientation of the transversus abdominis muscle fibers may similarly have influenced local anesthetic distribution, while the standing position and gravity may have favored ventral spread. However, this explanation remains speculative because injectate distribution was not assessed in vivo, and the cited evidence concerns intramuscular distribution of bupivacaine in human cadaveric tissue rather than interfascial distribution of lidocaine in horses. Future studies combining elastography with direct assessment of injectate distribution could help investigate whether tissue stiffness and anisotropy contribute to regional differences in sensory coverage. Such investigations could build on methods combining magnetic resonance elastography and diffusion tensor imaging to characterize anisotropic muscle mechanical properties [40]. Nevertheless, the dermatomal distribution observed in the present study should be interpreted with caution when extrapolating to clinical analgesic efficacy. Assessment of cutaneous sensory blockade remains the most widely accepted experimental approach for evaluating the extent of fascial plane blocks. However, increasing evidence suggests that the mechanisms underlying these techniques are more complex than simple dermatomal spread. Human studies have shown that cutaneous sensory loss does not always correlate with clinical analgesia, as interfascial plane blocks may preferentially affect nociceptive pathways without producing complete segmental cutaneous anesthesia [38,41]. Moreover, recent experimental evidence has demonstrated that local anesthetics may diffuse across fascial barriers beyond the distribution demonstrated by methylene blue or radiographic contrast, indicating that conventional cadaveric dye studies may underestimate the true biological spread of local anesthetics [39]. Although methylene blue distribution in the preliminary cadaveric dissection provided an anatomical guide for volume selection, it should not be interpreted as a direct representation of lidocaine spread in vivo.

Although the present TAP approach produced consistent abdominal wall desensitization, the distribution pattern suggests incomplete coverage of the more dorsal flank region where laparoscopic portals are commonly placed [26]. Desensitization at the coxal tuberosity level was minimal, indicating limited dorsal spread of local anesthetic. Therefore, this block may not provide complete analgesic coverage for all laparoscopic portal sites when used as a sole locoregional technique. However, the broader desensitization observed in the ventrocaudal abdominal wall may still be clinically relevant, particularly for reducing somatic nociceptive input associated with abdominal wall stretching and pneumoperitoneum during laparoscopic procedures [1,7].

Our findings partially align with previous equine TAP studies while also highlighting important differences related to injection approach and target region. Baldo et al. [23] reported extensive injectate spread after high-volume TAP administration in cadavers, although concerns regarding unintended femoral nerve involvement were raised. Freitag et al. [24] described a more ventral TAP approach targeting the lower abdominal wall, which may provide insufficient coverage of dorsally positioned laparoscopic portals. Similarly, Küls et al. [25] reported reduced responses to pinprick stimulation following TAP block, although block failure occurred in two of six horses, suggesting variability in effectiveness. Compared with these studies, the present approach was specifically designed for flank celiotomy and achieved sensory blockade in all horses, suggesting reliable interfascial spread within the targeted abdominal region.

Sedation was avoided to minimize pharmacological interference with sensory and motor assessments. However, stress during repeated testing may also influence behavioral responses. In clinical practice, low-stress handling and individually tailored sedation and perioperative analgesia remain important for horse comfort and the safety of both horses and personnel [2]. The unsedated experimental conditions used here should not be interpreted as a recommended clinical protocol.

Motor impairment occurred in seven of eight horses and was severe in two. Inadvertent proximal spread toward the caudal lumbar plexus, potentially affecting motor and proprioceptive fibers, represents a plausible anatomical explanation. Previous equine TAP investigations have raised concerns regarding unintended femoral nerve involvement [23]. Although cutaneous desensitization extended caudally to L5 in the present study, this does not establish involvement of the lumbar plexus or any specific nerve. Likewise, limited dorsal cutaneous desensitization does not exclude spread to deeper neural structures. Moreover, many nerves traversing interfascial planes are not purely sensory or motor but contain mixed sensory, proprioceptive and motor fibers [42], further complicating the interpretation of cutaneous sensory mapping. In addition, differential blockade depends on the local anesthetic, nerve fiber type, and concentration. Gokin et al. [43] demonstrated that Aδ sensory fibers and Aγ motor fibers were particularly susceptible to lidocaine blockade in the rat sciatic nerve, whereas C fibers required higher concentrations. This differs from the preferential blockade of C fibers described for bupivacaine and ropivacaine [38]. Concentration and dose may also influence motor effects. In dogs, epidural lidocaine 1% resulted in earlier recovery of ambulation than 2% administered at the same volume, without higher postoperative pain scores [44]. These findings support investigating whether lower lidocaine concentrations could reduce motor impairment while maintaining cutaneous desensitization following TAP block in horses. However, differential blockade was not assessed in the present study and remains a hypothetical contributor to the observed motor impairment. Alternatively, systemic absorption of lidocaine may also have contributed to the observed motor impairment. Recent pharmacokinetic data demonstrated measurable systemic absorption of bupivacaine following a rectus sheath block in horses [37], although comparable pharmacokinetic data for lidocaine administered within an interfascial plane are currently lacking. Although lidocaine and bupivacaine differ in their pharmacological properties, these findings highlight the need to better characterize the pharmacokinetic-pharmacodynamic relationships and toxic thresholds of local anesthetics following interfascial plane blocks in horses. None of these proposed mechanisms was directly evaluated. Injectate distribution in vivo, involvement of specific nerves or nerve fiber types, and plasma lidocaine concentrations were not assessed. Therefore, the relative contributions of local neural blockade and systemic absorption to the observed motor impairment cannot be determined, and these explanations should be regarded as hypotheses. Clinical translation also requires consideration of cumulative lidocaine exposure from TAP injections, skin infiltration, and any concomitant intravenous administration. Systemic absorption following abdominal interfascial blocks has been documented in humans, with plasma concentrations occasionally exceeding commonly accepted toxicity thresholds [45]. In a canine doctoral study of lateral quadratus lumborum block, different volume-concentration combinations delivering total bupivacaine doses of 2, 3, or 4.5 mg/kg were associated with increasing peak plasma concentrations, from approximately 973 to 2461 ng/mL. The highest-dose protocol was associated with prolonged clinical analgesic effects without observed clinical toxicity [46]. These findings illustrate the importance of the administered regimen when interpreting systemic exposure but cannot establish the pharmacokinetics or safety of lidocaine TAP blocks in horses. Consequently, the present findings do not establish the safety of combining this TAP protocol with intravenous lidocaine. Integration into balanced standing protocols would require evaluation of cumulative dosing and pharmacokinetics, together with the potentially additive motor effects of sedation and the block. As plasma lidocaine concentrations were not measured, the contribution of systemic exposure to the observed motor impairment cannot be determined. Further studies integrating anatomical, pharmacokinetic, and pharmacodynamic data are warranted to better elucidate the mechanisms underlying both sensory and motor effects of interfascial plane blocks in this species.

Several limitations should be considered when interpreting these findings. Cutaneous responses were assessed using a binary clamp-based method similar to that previously used in horses [25]. Although all assessments were performed by a single blinded investigator using a standardized technique, this method did not quantify mechanical sensory thresholds or the degree of partial desensitization. Each operator consistently performed injections on the same abdominal side. Therefore, operator and side effects could not be separated, and operator-related variability cannot be excluded. However, both operators were experienced in the technique, and correct needle-tip placement within the target interfascial plane was confirmed by ultrasound visualization of fascial separation during hydrodissection. Although this approach provides a reproducible and objective assessment of somatic sensory blockade under controlled conditions, it does not necessarily predict visceral analgesia or clinical analgesic efficacy. Visceral and referred pain may persist despite abdominal wall desensitization; therefore, if clinically validated, TAP block should be considered a component of balanced anesthesia and multimodal analgesia rather than a stand-alone technique for laparoscopy. Moreover, the study was performed in healthy horses under experimental conditions to allow standardized assessment of sensory and motor effects while minimizing potential confounding factors. Plasma lidocaine concentrations were not measured, and injectate spread was not confirmed in vivo. Therefore, the relative contributions of local and systemic effects to the observed sensory and motor responses could not be determined. Consequently, the clinical relevance of these findings for horses undergoing laparoscopic procedures remains to be established in prospective clinical trials. Future clinical studies could compare horses receiving TAP block with controls under otherwise standardized anesthetic protocols. Relevant outcomes could include intra-abdominal pressure, abdominal wall relaxation, ease of portal placement and instrument manipulation, surgical working conditions, supplemental analgesic and sedative requirements, and cardiorespiratory responses to pneumoperitoneum.

## 5. Conclusions

This ultrasound-guided two-point TAP block is a feasible and reproducible technique in standing horses and provides consistent desensitization of the lateral abdominal wall following lidocaine 2% administration. The block produced rapid onset and substantial multi-metameric sensory blockade, predominantly affecting ventrocaudal regions of the flank. However, the dorsal region showed limited coverage. Additionally, the frequent occurrence of transient motor impairment highlights the need for caution and further refinement of dosing and injection strategy. Future studies integrating clinical efficacy, pharmacokinetics, and safety assessment are warranted before clinical implementation.

## Figures and Tables

**Table 1:** Injection times (minute) recorded for the four transversus abdominis plan injections (right cranial, right caudal, left cranial, left caudal). Values are expressed as median (25th–75th percentiles).

Injection Point	Time (Minute)
Right cranial	2 (1–3)
Right caudal	3 (2–4)
Left cranial	2 (1–3)
Left caudal	2 (1–4)

## Data Availability

Data are available at https://github.com/v52mebaf/TAP-desensitization.git and https://github.com/v52mebaf/TAP-rest-of-data.git (accessed on 11 September 2026).

## Supplementary Materials

The following supporting information can be downloaded at: https://www.mdpi.com/article/10.3390/ani16213401/s1, Supplementary Material S1: ARRIVE Essential 10 checklist; Supplementary Material S2: Ultrasound-guided two-point transversus abdominis plane block technique in a standing horse.
